# Improved dose homogeneity using electronic compensation technique for total body irradiation

**DOI:** 10.1002/acm2.12316

**Published:** 2018-04-14

**Authors:** Tara E. Tyson, Matthew B. Podgorsak, Anurag K. Singh, Iris Z. Wang

**Affiliations:** ^1^ Roswell Park Cancer Institute State University of New York Buffalo NY USA

**Keywords:** electronic compensation, total body irradiation (TBI)

## Abstract

In total body irradiation (TBI) utilizing large parallel‐opposed fields, the manual placement of lead compensators has conventionally been used to compensate for the varying thickness throughout the body. The goal of this study is to pursue utilizing the modern electronic compensation (E‐comp) technique to more accurately deliver dose to TBI patients. Bilateral parallel‐opposed TBI treatment plans were created using E‐comp for 15 patients for whom CT data had been previously acquired. A desirable fluence pattern was manually painted within each field to yield a uniform dose distribution. The conventional compensation technique was simulated within the treatment planning system (TPS) using a field‐in‐field (FIF) method. This allows for a meaningful evaluation of the E‐comp technique in comparison to the conventional method. Dose–volume histograms (DVH) were computed for all treatment plans. The mean total body dose using E‐comp deviates from the prescribed dose (4 Gy) by an average of 2.4%. The mean total body dose using the conventional compensation deviates from the prescribed dose by an average of 4.5%. In all cases, the mean body dose calculated using E‐comp technique deviates less than 10% from that of conventional compensation. The average reduction in maximum dose using E‐comp compared to that of the conventional method was 30.3% ± 6.6% (standard deviation). In all cases, the s‐index for the E‐comp technique was lower (10.5% ± 0.7%) than that of the conventional method (15.8% ± 4.4%), indicating a more homogenous dose distribution. In conclusion, a large reduction in maximum body dose can be seen using the proposed E‐comp technique while still producing a mean body dose that accurately complies with the prescription dose. Dose homogeneity was quantified using s‐index which demonstrated a reduction in hotspots with E‐comp technique. Electronic compensation technique is capable of more accurately delivering a total body dose compared to conventional methods.

## 1 INTRODUCTION

In total body irradiation (TBI) the goal is to deliver a uniform dose to the patient's whole body.^1^ This serves to deplete the bone marrow and suppress the immune system of the patient as well as eliminate malignant cells.^2^ The uniform delivery of a total body dose encounters challenges that are not seen in more standard external beam radiation therapy procedures. In TBI the fields must exceed the scattering volume (patient's whole body) in all directions and the human body is irregularly shaped which makes achieving a uniform dose difficult. There have been a number of clinical setups and methods of compensation proposed to overcome these challenges.^3^, ^4^, ^5^, ^6^, ^7^, ^8^ A common treatment protocol is to use an unmodified standard linear accelerator and deviate from the standard room geometry to produce large parallel‐opposed beams. In our clinical setting, patients are positioned in a supine semi‐fetal position and irradiated bilaterally. The conventional method used to compensate for the varying thickness of a patient's body in the lateral direction is to manually place lead compensators on the treatment head. The number of layers of lead placed in a certain area of the treatment head is decided based on the lateral separation of the patient in the corresponding area. The monitor unit (MU) settings for this technique are typically chosen based on simple hand calculations. The treatment protocols of more commonly encountered diseases, such as breast cancer, have progressed significantly since the implementation of CT‐based treatment planning systems. This advancement has allowed for the capability to create a patient specific treatment plans which account for variables such as tumor size, shape, and tissue density. In contrast, the conventional treatment planning, setup, and delivery of TBI has yet to incorporate the advances of the field into a standard protocol. No procedural recommendations have been made since AAPM Report No. 17,^9^ almost 30 yr ago.

Electronic compensation is a forward planned technique that utilizes the dynamic MLC to deliver the beam and replaces the use of a mechanical compensator.^10^ It employs the use of the dynamic multi‐leaf collimator (dMLC) to achieve a homogeneous dose distribution when irradiating irregular surfaces. Within a standard application of electronic compensation, the “Irregular shape compensator” module within the Varian ECLIPSE treatment planning platform (version 10 with AAA 10.0.28; Varian Medical Systems, Palo Alto, CA, USA) is used to calculate the optimal field fluence based on CT data. This function calculates the fluence such that a homogeneous dose is delivered to a user specified depth. Once the optimal fluence is calculated, it is converted into a deliverable fluence and then translated into MLC leaf motion by the leaf motion calculator (LMC).^10^

We present here a comparison of our novel electronic compensation method to simulated conventional compensation for the delivery of a total body dose. By combining multi‐leaf collimator with a three‐dimensional treatment planning system, our work seeks to produce a dynamic photon beam that can generate an optimized fluence pattern that results in a more uniform TBI dose distribution. This research aims to demonstrate that modifications to typical electronic compensation implementations can allow the technology to be adapted to the constraints of TBI and improve upon dosimetric accuracy of treatment delivery in comparison to standard TBI compensation methods.

## 2 MATERIALS AND METHOD

### 2.A General procedure

Fifteen previously treated patients were selected for whom CT data, from head to mid‐calf, had been previously acquired. These patients show a large range of lateral thorax separation (43.3–63.8 cm). Four bilateral TBI treatment plans, with a total prescription dose of 4 Gy administered in two fractions, BID, to the midplane at the umbilicus level, were generated for each patient using the Varian treatment planning system (TPS) at extended SAD of 377 cm: (a) conventional compensation with tissue density correction (TDC) off, (b) conventional compensation with TDC on, (c) electronic compensation with TDC off, and (d) electronic compensation with TDC on. All plans were generated using a 6 MV photon beam. The plans calculated with the heterogeneity correction off are used to imitate the simple monitor unit (MU) calculation traditionally performed for TBI. The plans generated with the tissue density correction on were calculated utilizing the preset MU values from the plans with TDC off for comparison. This yields a more accurate representation of the dose distributions within a heterogeneous body when the MUs are calculated without considering a variation in tissue density.

### 2.B Conventional compensation simulated in TPS

**Figure 1:**
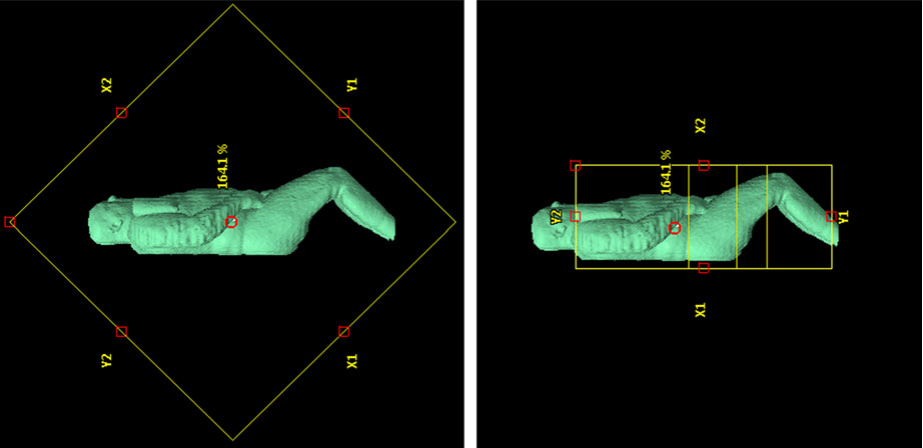
Conventional simulation fields: Beams eye view of bilateral TBI treatment fields for patient 13. A large open field with a collimator angle of 45°and a succession of smaller fields with a collimator angle of 0°.

**Figure 2:**
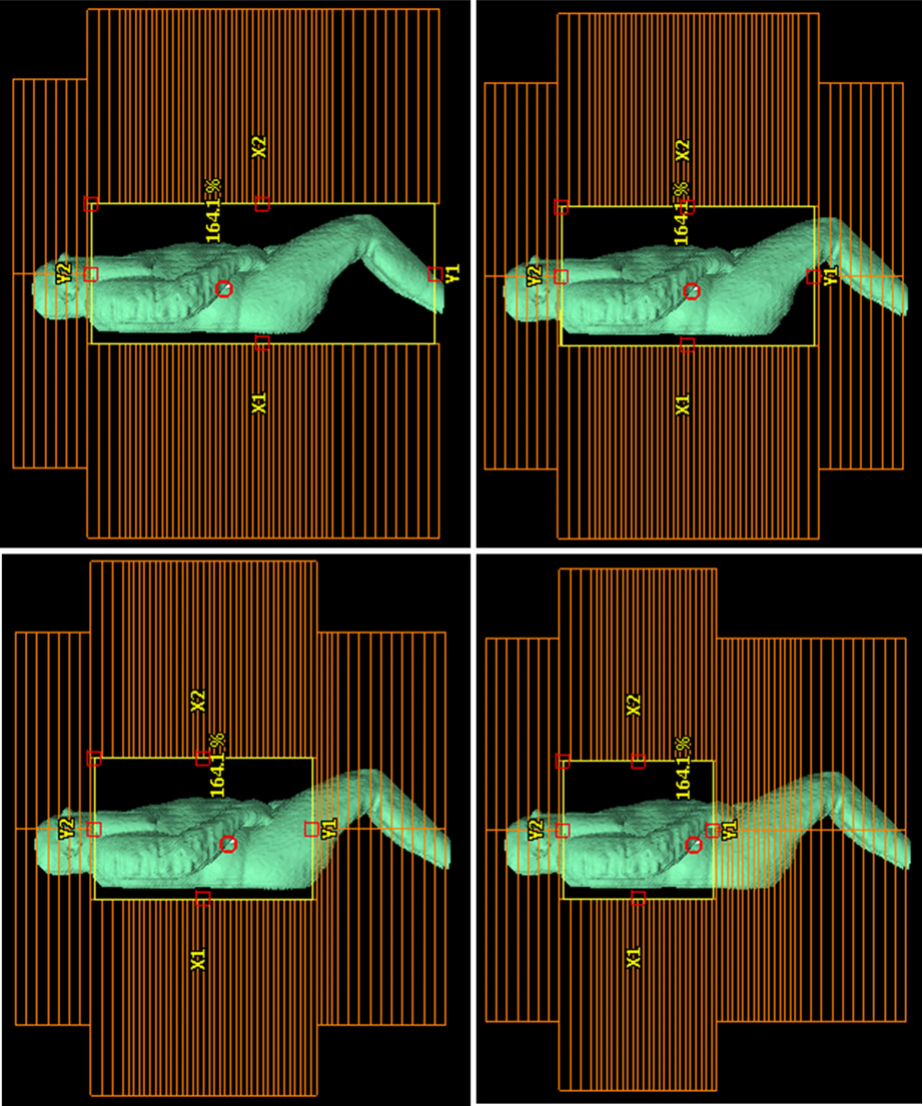
MLC configuration: Beam's eye view of the MLC leaves forming the succession of smaller fields for patient 13.

For the purpose of comparison, the conventional method was simulated using the Varian treatment planning system. Treatment plans were created retrospectively using a field‐in‐field (FIF) technique, with the multi‐leaf collimator (MLC) mimicking the effect of the lead compensation. Treatment fields include: one pair of large open fields with the collimator set to 45° and a succession of smaller paired fields with the collimator angle set to 0° (Fig. 1). The smaller fields are shaped by moving MLC which blocks sections of the patient once they have received close to 100% of the prescribed dose (Fig. 2).

Each field was assigned a specific weight which was calculated based on the number of layers of lead used to compensate for that particular bilateral separation of the specific patient at the time of conventional delivery. With the knowledge of the linear attenuation coefficient of the lead compensators, the transmission was calculated for each differential thickness of lead. For patient 13 the lead compensators had an attenuation coefficient of 0.05406 and the transmission was calculated for five different thicknesses of lead (Table 1). Once the desired transmission was calculated for each field, it was used to determine the weight which would be assigned in the TPS. A field weight of 1.0 signifies that the field is open throughout the entire treatment and 0.0 would indicate that it is blocked throughout the treatment. In this study, the fields are executed in succession from the largest to the smallest with each of the smaller fields encompassed by the previous larger field. Beginning with the smallest field, which has a transmission of 1.0, the weight is determined by subtracting the transmission of the next largest field. For patient 13 the smallest field is the shoulder/umbilicus (transmission = 1.0) and the next largest field is the pelvis (transmission = 0.828) which would yield a field weight of .172 (1.0 − 0.828 = 0.172). This process is continued until the last and largest field is reached. For patient 13 the largest field is the Open field (transmission = 0.552) and there is no subsequent field therefore 0.0 is subtracted yielding a field weight of 0.552. The sum of the weights from each field equals one in all cases.

**Table 1:** Calculation of field weight for patient 13

Patient 13					
Field name	No. of layers of Pb	Thickness (mm)	Attenuation coefficient (Pb)	Transmission	Weight
Head/Neck/Ankle	22	11	0.05406	0.552	0.552
Knee/Calves	15	7.5	0.05406	0.667	0.115
Thigh	8	4	0.05406	0.806	0.139
Pelvis	7	3.5	0.05406	0.828	0.022
Shoulder/Umbilicus	0	0	0.05406	1.000	0.172

### 2.C Electronic compensation for TBI

**Figure 3:**
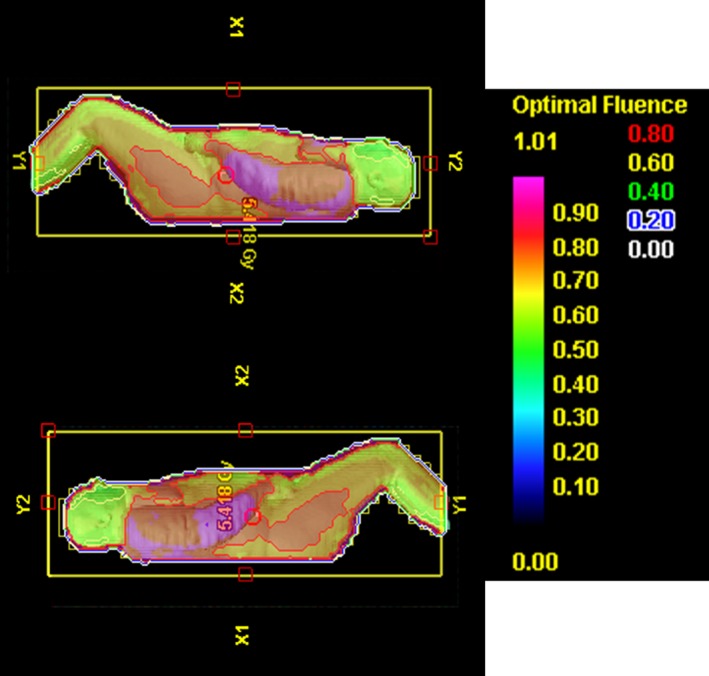
Optimal fluence beams eye view of the manually painted optimal fluence pattern for patient 13.

Because of the extended distance at which TBI is delivered, and the necessary extension of the field beyond scattering volume in all directions, the current Eclipse^TM^ TPS module cannot calculate the optimal fluence as it does for cases that have a more standard geometry. Therefore, a desirable fluence pattern was manually painted within each field for each patient (Fig. 3). To achieve an appropriate fluence pattern, a pair of large open fields was delivered without any compensation and TDC using the CT data from the patient. The isodose lines produced by these uncompensated fields are an indication of the compensation needed to produce a uniform dose distribution. The isodose lines were first converted to structures, using the function in the TPS, and used as a map for manually painting the fluence. The isodose structures indicate what transmission coefficient should be used in that area of the anatomy Starting with the highest dose and working down in 10% increments until 100% is reached at which point the entire target (whole body structure) is present. For patient 13, the highest dose seen was 180% of the prescription dose. The transmission coefficient used to paint each new structure was simply calculated by dividing the desired dose (100%) by the dose received without compensation. For example, the 180% isodose structure for patient 13 was painted with the transmission coefficient 0.556 (100%/180%) before moving on to the 170% isodose structure. The dynamic motion of the MLC executes the delivery of the painted fluence distribution.

### 2.D Comparison of electronic vs conventional compensation

**Figure 4:**
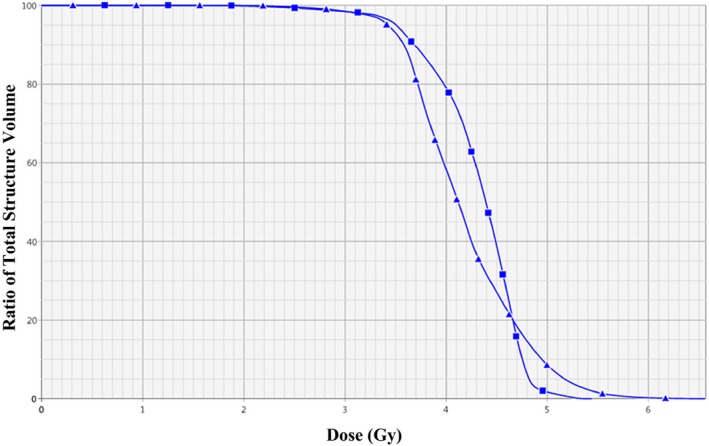
Cumulative DVH of mean body dose cumulative DVH of the dose within the body of patient 13. The line with squares represents the body dose for the plan that utilized the electronic compensation method. The line with triangles represents the body dose for the plan that simulates the conventional treatment method.

The conventional planning and delivery techniques of TBI were compared to the application of electronic compensation to TBI delivery. Cumulative dose–volume histograms (DVH) were generated to qualitatively compare the two methods (Fig. 4). The mean total body dose and maximum dose within the body were recorded for all four plans for each case. The s‐index was used to quantitatively evaluate the merit of each technique. The s‐index is a dose–volume histogram (DVH)‐based homogeneity index that is defined as the standard deviation of the normalized differential DVH (dDVH) curve.^11^ The dDVH is a plot of the volume receiving a dose within a specified dose range. Although less commonly used than the integral DVH curve, the differential DVH curve can be useful in that it can provide information regarding the extent of dose variation within a structure. The standard deviation of the dDVH curve quantifies the spread of the average giving an indication the inhomogeneity of the dose. A larger standard deviation of the dDVH curve correlates to a more sizeable spread and, therefore, a less homogeneous dose distribution.^11^

## 3 RESULTS AND DISCUSSION

### 3.A Materials minimal variation in mean body dose — TDC on vs TDC off

**Figure 5:**
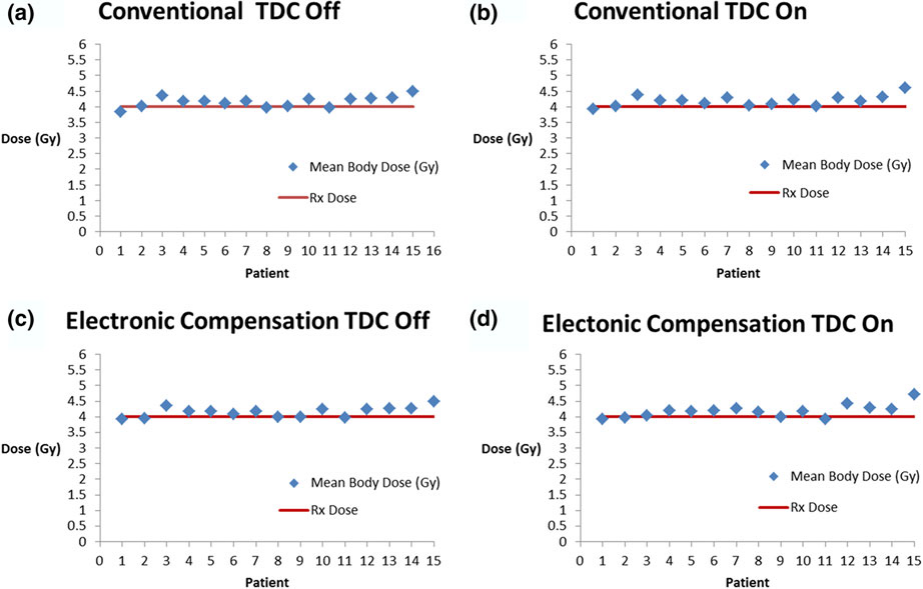
Mean body dose for each patient The solid line in each graph represents the prescription dose, which in each case is 4 Gy. The mean body dose for each patient utilizing (a) the simulation of the conventional method of TBI with TDC off (b) the simulation of the conventional method with TDC on (c) the electronic compensation technique with TDC off (d) the electronic compensation technique with TDC on.

With respect to mean body dose, both the conventional simulation and the electronic compensation technique, with and without heterogeneity corrections, were able to deliver the prescription dose (4 Gy) with good accuracy as seen in Fig. 5. In all cases, the mean body dose from plans using electronic compensation deviates less than 7.1% from the prescription dose.

Within this study, a distinction was made between plans that were calculated with the heterogeneity correction (TDC) on and those with it off. This is because the MU calculations for TBI are traditionally performed without accounting for the varying tissue density within a patient. When these MU calculations are used to deliver a whole body dose to a heterogeneous body, this will cause a misrepresentation of the actual dose distribution. This can be seen in the plans calculated with TDC off.^12^ These MU calculations (with TDC off) were then used as preset values when the TDC was on. This generated dose distributions that are a more accurate representation of the conventional planning and delivery of TBI within a heterogeneous body. In the study conducted by Bailey, this technique was used to show the significance of tissue density corrections for bilateral TBI lung doses. For consistency, this technique was also employed for the calculation of plans using electronic compensation. In all cases, very minor deviations exist when comparing the mean total body dose with and without TDC as well as the maximum body dose with and without TDC. The insignificance of these deviations suggests that, unlike lung dose calculations, the consideration of varying tissue densities (other than lung) is inconsequential to the mean total body dose and maximum body dose. Therefore, from this point forward all comparisons discussed will be done between the conventional simulation with TDC on and the electronic compensation with TDC on.

### 3.B Dosimetric comparison of electronic vs conventional compensation

**Figure 6:**
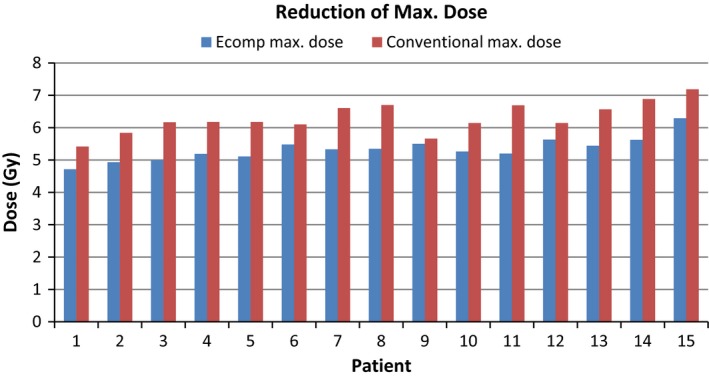
Maximum body dose for each patient graph show the reduction of the maximum body dose in each case evaluated in this study with the use of the electronic compensation technique.

There was an evaluation of the electronic compensation technique in comparison to the conventional simulation. In every plan that was generated in this study, the maximum body dose was reduced using the electronic compensation technique (Fig. 6). The average reduction in maximum dose was 30.3% with a standard deviation of 6.6% (Table 2). The mean total body dose for the plans utilizing the electronic compensation technique remained within 10% of the mean body dose for the conventional simulation (Table 3). As seen in Table 3, the mean total body dose using electronic compensation deviates from the prescribed dose (4 Gy) by an average of 2.4%. The mean total body dose in the plans simulating the conventional compensation deviates from the prescribed dose (4 Gy) by an average of about 4.5%.

**Table 2:** The maximum body dose seen in both the electronic compensation technique and the conventional simulation for each patient. As well as the reduction of maximum body dose

Patient	Bilateral thorax separation (cm)	Max. dose _CONV._ (% of Rx dose)	Max. dose _Ecomp._ (% of Rx dose)	Reduction of max. dose (% of Rx dose)
1	43.3	135.4	115.0	20.4
2	48.2	146.0	121.7	24.3
3	47.8	144.9	114.9	30.0
4	41.7	154.5	121.3	33.2
5	49.1	156.7	120.1	36.6
6	51.1	152.5	130.8	21.7
7	47.8	165.2	125.9	39.3
8	53.9	167.5	135.0	32.5
9	51.9	141.5	137.4	18.1
10	52.5	153.6	123.7	29.9
11	53.0	167.3	133.9	33.4
12	55.6	153.6	126.7	26.9
13	61.0	164.1	124.6	39.5
14	55.3	172.2	141.1	31.1
15	63.8	179.7	141.6	38.1
Average		157.0	127.6	30.3
Std. Dev.		11.8	8.4	6.6

**Table 3:** The mean body dose for the simulation of the conventional technique and electronic compensation

Patient	Bilateral thorax separation (cm)	Mean dose _Conv._ (% of Rx. Dose)	Deviation from Rx. dose (%)	Mean dose _Ecomp_ (% of Rx. Dose)	Deviation from Rx. dose (%)
1	43.3	98.0	2.0	95.6	4.4
2	48.2	101.0	1.0	99.8	0.2
3	47.8	102.8	2.8	93.0	7.0
4	41.7	104.9	4.9	99.3	0.7
5	49.1	101.4	1.4	99.0	1.0
6	51.1	102.8	2.8	100.1	0.1
7	47.8	107.1	7.1	100.8	0.8
8	53.9	101.2	1.2	104.7	4.7
9	51.9	102.1	2.1	99.9	0.1
10	52.5	106.9	6.9	98.0	2.0
11	53.0	100.2	0.2	101.0	1.0
12	55.6	107.1	7.1	99.4	0.6
13	61.0	104.5	4.5	98.5	1.5
14	55.3	108.0	8.0	106.5	6.5
15	63.8	115.0	15.0	106.0	6.0
Average		104.2	4.5	100.1	2.4
Std. Dev.		4.0	3.8	3.4	2.4

**Figure 7:**
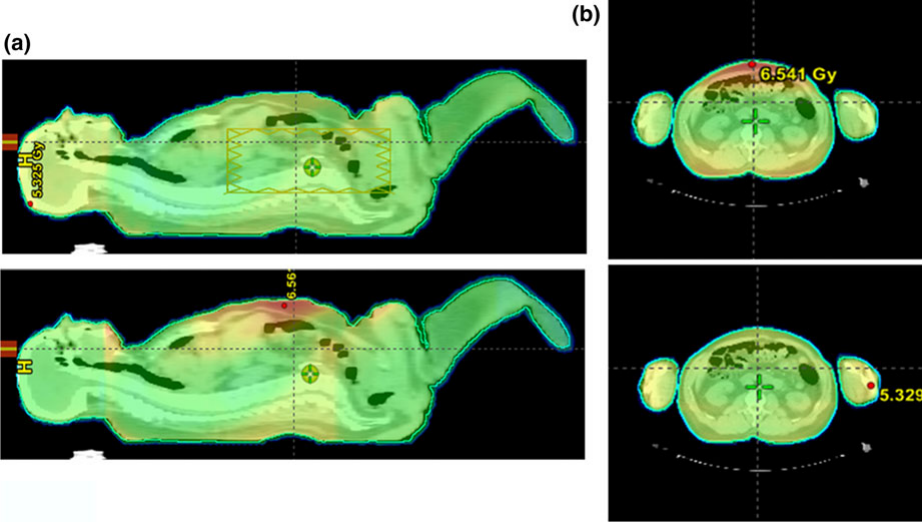
Hotspot in anterior abdomen (a) The dose distribution for patient 13 at the midsagittal plane for the conventional simulation on the left and the electronic compensation technique on the right. (b) The dose distribution for patient 13 in a transverse plane at the umbilicus level. The conventional simulation is shown above and the electronic compensation is pictured below.

An important result of this study is the reduction in maximum body dose that is seen with the use of the electronic compensation technique for bilateral TBI. The goal of TBI is uniform dose distribution that is as close to the prescription dose as possible.^1^ Any part of the body that receives a significantly higher dose than the prescribed 4 Gy, is considered a hotspot. Therefore the reduction in the size and intensity of hotspots is a desired effect of a new delivery technique. For every patient investigated within this study, the maximum body dose was significantly reduced with the use of electronic compensation. In the plans that simulate conventional delivery technique, the “hotspots” are typically found in the portion of the abdomen most anterior to the umbilicus, as seen in Fig. 7. This is a direct result of two‐dimensional compensation. The amount of radiation received by this segment of the patient is based solely on the bilateral separation at midplane of the umbilicus without taking into consideration the tapering of the separation of the abdomen anterior to the midplane. With the use of electronic compensation, the amount of radiation received by any part of the patient is based on desired fluence in that particular area. The reduction in the size of the abdominal hotspot when using electronic compensation can be seen qualitatively in Fig. 7. These results suggest that the dose distribution for the electronic compensation technique is more homogenous than that of the conventional simulation.

### 3.C Quantitative S‐index comparison of electronic vs conventional compensation

**Figure 8:**
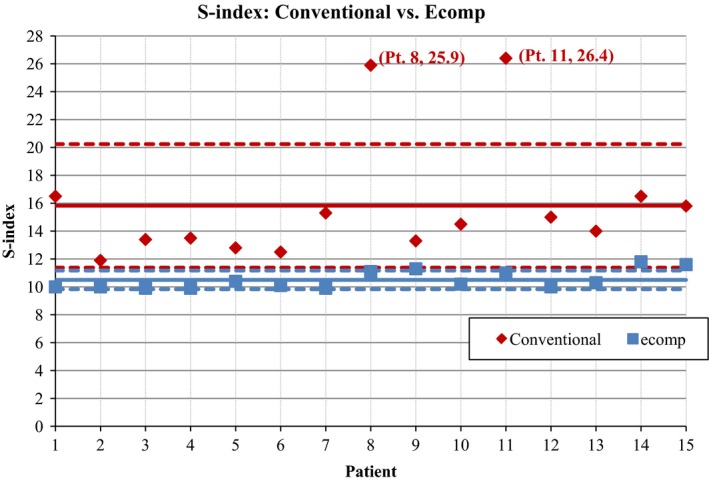
s‐index plotted for each patient The red diamonds represent the s‐index for each patient using the simulation of the conventional method. The blue squares represent the s‐index for each patient using the electronic compensation technique. The solid lines represent the mean s‐index and the dashed lines indicate the standard deviation for each set of data.

**Figure 9:**
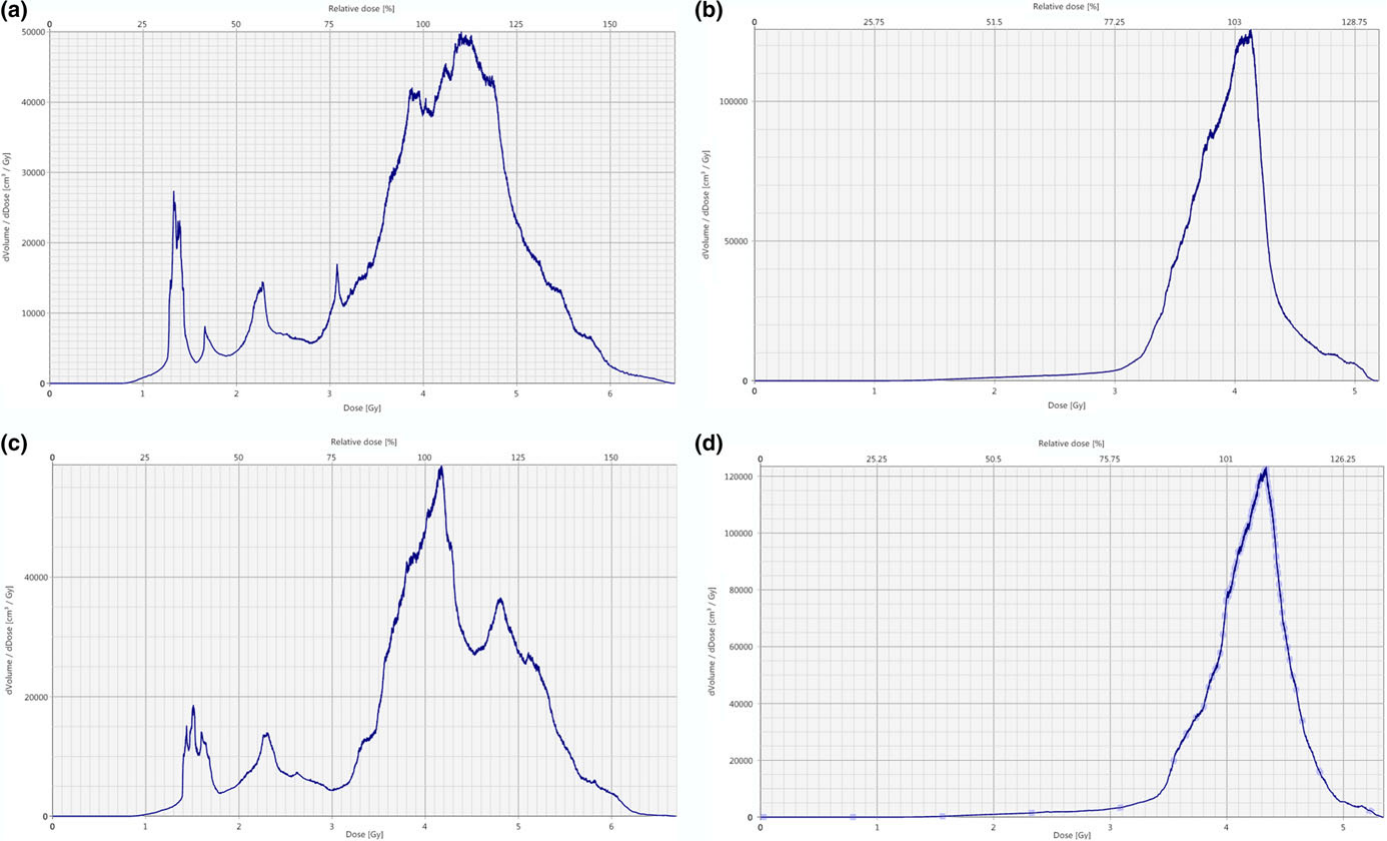
dDVH Patient 8 conventional dDVH (a) and the dDVH for the electronic compensation technique (b). Patient 11 conventional dDVH (c) and the dDVH for the electronic compensation technique (d). The *x*‐axis represents Dose (Gy) and the *y*‐axis represents dVolume/dDose (cm^3^/Gy).

To quantify the dose homogeneity, the s‐index was used for comparison between electronic and conventional compensation for TBI. In all 15 cases that were analyzed, the s‐index for the electronic compensation technique was lower, indicating a more homogenous distribution (Fig. 8). The average s‐index for the conventional simulation was 15.8% with a standard deviation of 4.4%. The average s‐index for the electronic compensation was 10.5% with a standard deviation of 0.7%. In Fig. 8, the solid lines represent the mean s‐index for each technique and the dashed lines show the standard deviation for each set of data. There are two patients for whom the s‐index of the conventional simulation is significantly higher than for any other patients. This indicates a poor homogeneity within the dose distribution for these plans. This is confirmed when we consider the DVH for each of these plans (Fig. 9).

### 3.D Qualitative comparison of electronic vs conventional compensation

The electronic compensation technique would improve the accuracy with which we are able to deliver a prescribed total body dose. The conventional method relies on the ability to match a shadow that is projected onto the patient, using the light field and lead compensation, to the corresponding section of the anatomy that is planned to receive that amount of compensation. This could prove to be particularly difficult in case of the neck/shoulder juncture. From the lateral view it is hard to distinguish the exact point at which the neck ends and the shoulders begin. This could have dangerous results due to the drastic difference in lateral separation of these two parts of the patient anatomy. For example, the compensation used for patient 13 in the conventional delivery required 22 layers of lead to shield the neck and 0 layers of lead to shield the shoulders (Table 1). If this compensation was placed slightly inferior to the planned position, it could block part of the shoulder causing the under dosing of the shoulders and mediastinum. If it is placed superior to the planned position it would not shield all of the neck and therefore could create a hotspot in an area with such small lateral separation. The electronic compensation technique is far less prone to human error because all compensation is carried out by the movement of MLC leaves during the treatment. This method of compensation can have quality assurance protocols in place to ensure the machine is able to carry out the plan with a high level accuracy.

## 4 CONCLUSION

We have proposed a new method for the planning and delivery of bilateral total body irradiation using electronic compensation. The most remarkable difference between electronic compensation and conventional methods with regard to maximum body dose; an average 30% reduction in maximum body dose can be observed with our proposed electronic compensation technique. Dose homogeneity was quantified using s‐index and demonstrated that electronic compensation can reduce hotspots in comparison to conventional compensation. This study also indicates that the use of electronic compensation in the planning and delivery of TBI provides a mean total body dose that more accurately complies with the prescribed dose. In summary, this study illustrates that an electronic compensation technique is capable of more accurately delivering a total body dose compared to current conventional methods.

## CONFLICT OF INTEREST

The authors declare no conflict of interest.
